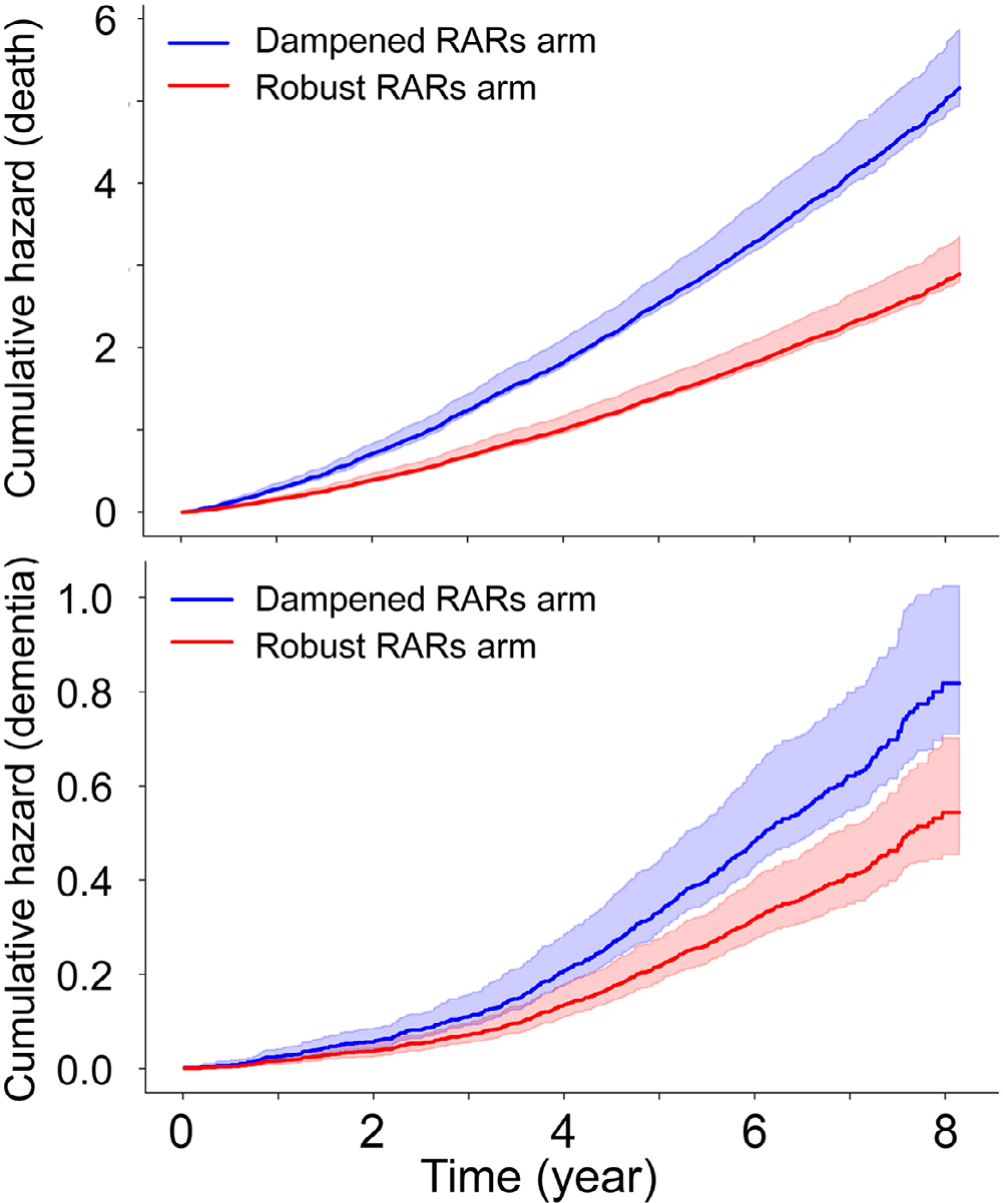# Target trial emulation analysis linking dampened circadian rest‐activity rhythms and dementia

**DOI:** 10.1002/alz.092574

**Published:** 2025-01-09

**Authors:** Haoqi Sun, Jingyun Yang, Ricardo A Vialle, Chenlu Gao, Ruixue Cai, Shahab Haghayegh, Lei Gao, Martin K Rutter, Kun Hu, Peng Li

**Affiliations:** ^1^ Beth Israel Deaconess Medical Center, Boston, MA USA; ^2^ Rush University Medical Center, Chicago, IL USA; ^3^ Rush Alzheimer’s Disease Center, Chicago, IL USA; ^4^ Rush Alzheimer’s Disease Center, Rush University Medical Center, Chicago, IL USA; ^5^ Massachusetts General Hospital, Boston, MA USA; ^6^ Harvard Medical School, Boston, MA USA; ^7^ Broad Institute of MIT and Harvard, Cambridge, MA USA; ^8^ University of Manchester, Manchester United Kingdom

## Abstract

**Background:**

Previous studies have linked disrupted circadian rest‐activity rhythms (RARs) with increased risks for Alzheimer’s disease and related dementias. However, these findings may be confounded due to the observational nature and offer limited insights into causality. We aimed to employ target trial emulation to estimate potential causal effects of RARs on dementia development.

**Method:**

Using the UK Biobank data, we emulated a target randomized trial including 86,802 participants with ≥6 days of actigraphy. Cycle‐by‐cycle amplitudes of the ∼24‐h RARs were computed from actigraphy using uniform‐phase empirical mode decomposition. Two emulated trial arms were defined (1) a ‘robust RARs’ arm within which all participants had higher than cohort median amplitudes averaged over six consecutive days, and (2) a ‘dampened RARs’ arm where all participants had lower (than cohort median) values in six consecutive days. The primary outcome was time to all‐cause dementia, and the secondary outcome was time to death. Covariates considered included age, sex, race, college education (yes/no), body mass index, socioeconomic status (Townsend deprivation index), number of active medications, history of circulatory (yes/no), nervous system (yes/no), and mental or behavioral health conditions (yes/no). In the emulated trial, the baseline was defined as the beginning of actigraphy. The g‐formula and cause‐specific Cox proportional hazards model, complemented by bootstrapping for 95% confidence intervals (CI), were used for analysis.

**Result:**

Over an 8‐year follow‐up, 467 participants developed all‐cause dementia, and 2,769 died. The ‘dampened RARs’ arm exhibited a significantly higher risk of death than the ‘robust RARs’ arm. Also, a higher cumulative incidence of dementia was observed in the ‘dampened RARs’ arm after accounting for death as a competing risk. At 8 years post‐baseline, the cumulative incidence of dementia was 0.84% (95% CI: 0.71%–1.1%) in the ‘dampened RARs’ arm, versus 0.56% (0.46%–0.70%) in the ‘robust RARs’ arm (i.e., dampened RARs lifestyle had a 1.5‐fold increased risk compared to a robust RARs lifestyle).

**Conclusion:**

The findings from this multivariable‐adjusted target trial emulation suggest that perturbed RARs may be causally associated with increased risk of all‐cause dementia. These results underscore the importance of maintaining robust RARs for dementia prevention.